# Metformin-associated severe lactic acidosis combined with multi-organ insufficiency induced by infection with *Aeromonas veronii*: A case report

**DOI:** 10.1097/MD.0000000000032659

**Published:** 2023-01-13

**Authors:** Yu Xia, Xiaofeng Zhu, Changxue Wu

**Affiliations:** a Department of Anesthesiology, The Affiliated Hospital of Southwest Medical University, Luzhou, China; b Department of Critical Care Medicine, The Affiliated Hospital of Southwest Medical University, Luzhou, China; c Department of Anesthesiology, School of Clinical Medicine, Southwest Medical University, Luzhou, Sichuan Province, China.

**Keywords:** *Aeromonas veronii*, diabetes, lactic acidosis, metformin

## Abstract

Lactic acidosis is a disease in which lactic acid accumulates in the blood and causes acidosis in the patient. The criteria for diagnosis are a lactate level of >2 mmol/L in the blood and a blood pH of <7.2.

**Patient concerns:**

A 72-year-old Asian female with a history of diabetes for 20+ years was admitted to the hospital with the chief complaint of “dry mouth, polydipsia for 20+ years, loss of appetite for 5+ days, vomiting for 1-day.” She was admitted with a blood gas pH of 6.795, and a lactate level of >30 mmol/L.

**Diagnoses:**

Type 2 diabetes mellitus with lactic acidosis, ketoacidosis, chronic renal insufficiency, hypertensive disease, and coronary arteriosclerotic heart disease.

**Interventions:**

She was treated with symptomatic rehydration and ketone reduction immediately, but then became unconscious and was admitted to the intensive care unit, where she was administered symptomatic support and continuous renal replacement therapy. As the blood culture showed *Aeromonas veronii*, she was administered a sensitive antibiotic in conjunction.

**Outcomes:**

However, after achieving a stable internal environment and good infection control, the patient’s family decided to discontinue treatment because of persistent heart failure with acute exacerbation of chronic renal insufficiency complicated by gastrointestinal bleeding.

**Lessons:**

Lactic acidosis has low incidence, poor prognosis, and high morbidity and mortality rates. Special attention should be paid to infection-induced acidosis, especially in patients with combined multi-organ insufficiency. Early diagnosis and active management can improve the patient prognosis.

## 1. Introduction

Aeromonas is a gram-negative anaerobic bacilli.^[1,2]^ *Aeromonas veronii* (*A veronii*) is 1 of 27 species of *Aeromonas*.^[3,4]^ They are ubiquitous in food and soil. *Aeromonas* is responsible for several human infectious diseases such as gastroenteritis, wound infections, hepatobiliary infections, necrotizing fasciitis, and sepsis.^[5]^ Humans carry *Aeromonas* in their gastrointestinal tract, and the carriage rate of *Aeromonas* in the feces of healthy individuals ranges from 0% to 4%.^[6]^ Several infections caused by *A veronii* are self-limiting. However, in patients with severe underlying disease or in individuals with underlying disease or immunocompromised individuals, invasive infections may be urgent and rapidly progressive. In recent years, an increasing number of studies have shown that *A veronii* has become an important human, animal, and aquatic pathogen,^[7,8]^ that can cause gastroenteritis, peritonitis, sepsis, and traumatic infections in humans,^[9]^ posing a serious threat to human health, especially to the elderly, children, and immunocompromised populations.^[10–13]^ Studies have shown that *A veronii* may cause gastroenteritis through several processes: first, through the acidic environment of gastric juice with the help of the bacteria’s own acid tolerance, directed movement and adsorption to the epithelial cells of the gastrointestinal tract, formation of biofilm and colonization of the intestine, and finally release of virulence factors and initiation of infection.^[1,14]^ Metformin, the drug of choice for the treatment of type 2 diabetes, occupies a leading position in domestic and international treatment guidelines.^[15]^ It has been considered a safe hypoglycemic agent for many years. Lactic acidosis is a disease in which lactic acid accumulates in the blood and causes acidosis in the patient. The criteria for diagnosis are a lactate level of >2 mmol/L in the blood and a blood pH of <7.2.^[16,17]^

## 2. Case presentation

An elderly female, 72 years old, was admitted to the Department of Endocrinology with complaints of “dry mouth, polydipsia” for 20+ years, loss of appetite for 5+ days, vomiting for 1-day. “She had a history” of diabetes mellitus for 20 years, and was taking metformin regularly for long-term glucose-lowering. Previous comorbid hypertension for >4 years and renal insufficiency for >1 year.

**Figure 1.**
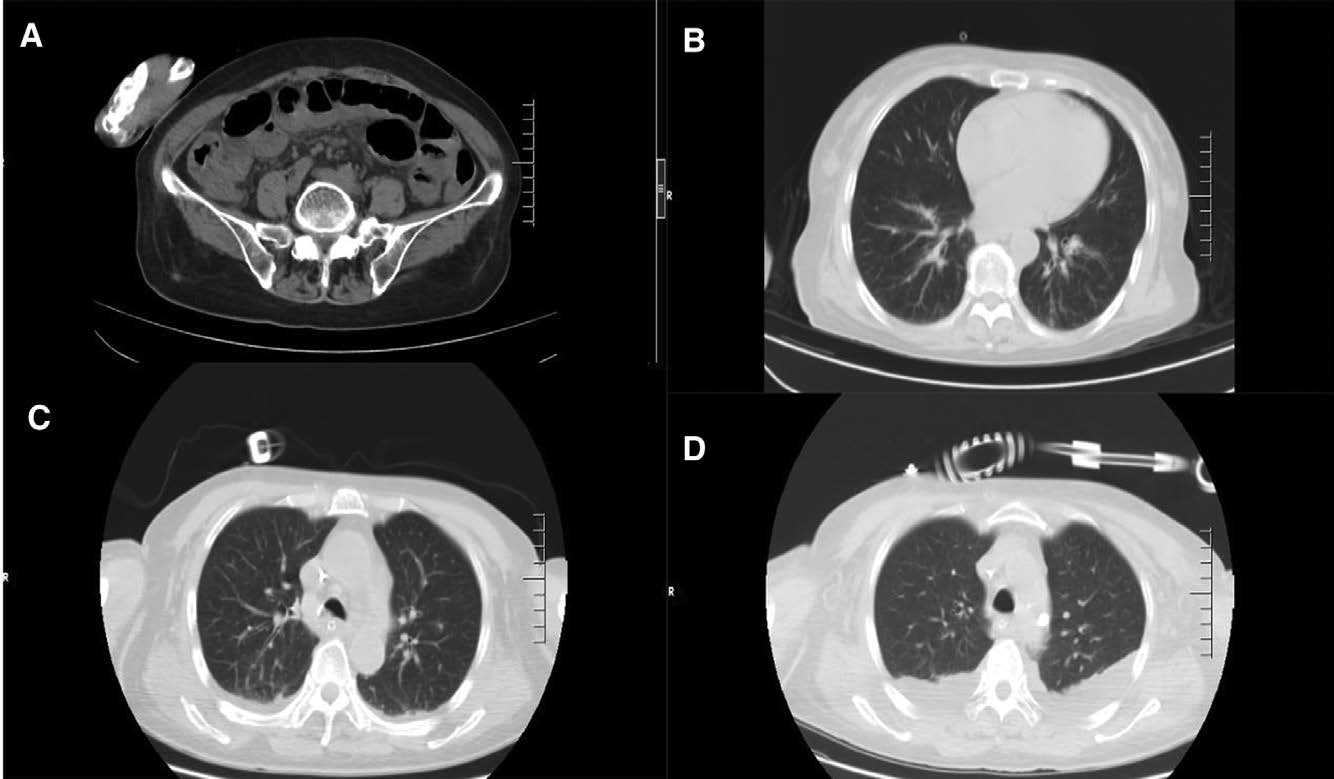
CT of the chest and abdomen since admission. (A) Abdominal CT on July 11 showed that part of the small intestine was dilated, fluid accumulation, gas accumulation, and gastric cavity was dilated. (B) Chest CT on July 11 showed no obvious inflammation and other abnormalities in the lungs. (C) Chest CT on July 15 showed a small bilateral pleural effusion. (D) Chest CT on July 19 showed partial external pressure atelectasis with bilateral pleural effusion. CT = computed tomography.

First admission blood gas: pH 6.795, PO2t 152.7 mm Hg, PCO2t 13.4 mm Hg, BE 30.26 mmol/L, BEecf 32.58 mmol/L, AG 40.70 mmol/L, blood lactate lac > 30 mmol/L, blood ketone 4.7 mmol/L. Electrocardiogram: sinus tachycardia; ST-T changes. The results of chest and abdominal computed tomography examinations during admission and intensive care unit (ICU) treatment are shown in Figure 1. The changes in each index that reflect the function of various organs during ICU treatment are shown in Table 1.

**Table 1:** Summary of the laboratory test.

Time	Day 1	Day 2	Day 4	Day 8	Day 11	Day 14	Day 17	Day 18
PCT (ng/m**L**)	3.32	5.51	26.34	5.85	5.05	8.86	10.46	7.62
White **blood** cell (×10^9^/L)	28.63	23.98	23.7	9.75	35.3	26.65	32.85	36.26
Neutrophil **ratio** (%)	82.8	91.4	90.7	75.9	85.7	94.5	91.9	94.4
Creatinine (µmol/L)	708.8	670.3	101.5	357.8	362.9	429.6		
GFR (m**L**/min)	4.5	4.9	47.6	10.3	10.1	8.3		
ALT (U/L)	28.8	37.3	68.6	14.7	9.4	5		
AST (U/L)	106.8	146.4	179.6	50	73.7	65.8		

ALT = alanine transaminase, AST = aspartate aminotransferase, GFR = glomerular filtration rate, PCT = procalcitonin.

Diagnoses: Type 2 diabetes mellitus with lactic acidosis; ketoacidosis; chronic renal insufficiency; hypertensive disease; and coronary arteriosclerotic heart disease? (CAD) She became unconscious at 21:25 on July 11, 2022 and was transferred to the ICU at 23:15 for further treatment. She was in a comatose state, with severe acidosis and deep breathing, and was immediately intubated. She was given meropenem 1 g q8h anti-infection treatment. Because of severe metabolic acidosis, the renal creatinine was 708.8 μmol/L(normal 44–133 μmol/L), glomerular filtration rate was 4.5 mL/minutes(normal 80–125 mL/minutes), BNP was 6714.00 pg/mL(normal 0–38 pg/mL). This patient was considered to have cardiac and renal insufficiency and was given continuous renal replacement therapy.

**Figure 2.**
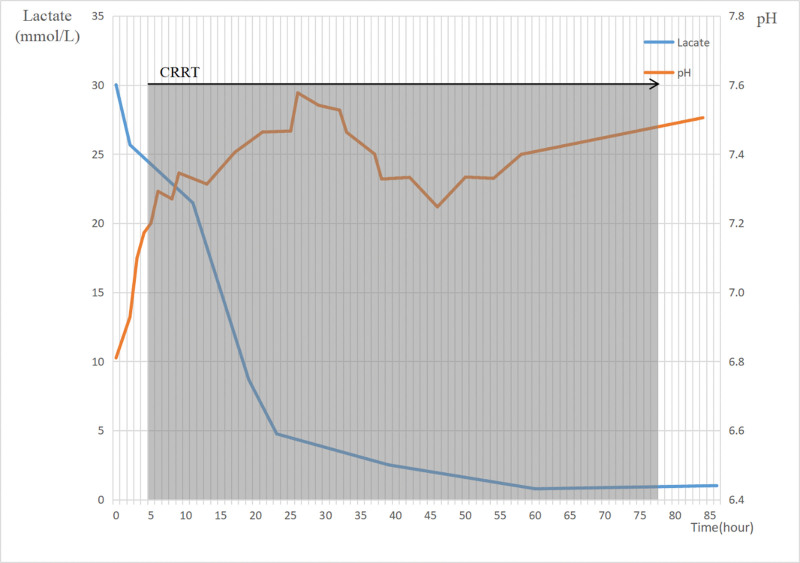
The clinical time course of lactate level and pH.

After 2 days of active rehydration, acid correction, pumping insulin to lower glucose and ketones, and bedside dialysis treatment, the patient’s lactate was rechecked at 0.78 mol/L. It decreased to the normal level, and disturbance of the internal environment was corrected; therefore, continuous renal replacement therapy was discontinued. The blood culture report suggested *A veronii*, therefore meropenem 1 g q8h was changed to cefoperazone sodium sulbactam 3 g q8h combined with ornidazole 1 g q12h, which was sensitive to the drug sensitivity test. After changing the antibiotics, the patient’s infection index was controlled and the temperature was not febrile. Symptoms of anemia and gastrointestinal bleeding began to appear on the 10th day of hospitalization, and renal function continued to decline. Continuous renal replacement therapy was administered again, red blood cell suspension and fresh frozen plasma were transfused, and continuous anti-infective and symptomatic supportive treatment was performed. Unfortunately, the patient’s family chose to abandon treatment on July 28, 2022. The pH and lactate levels of the patient during hospitalization are shown in Figure 2. The patient’s timeline since admission is shown in Table 2.

**Table 2:** Time line since the patient was admitted.

Timeline	Patient condition
Before admission	“dry mouth, polydipsia for 20+ years, loss of appetite for 5+ days, vomiting for 1-day”
D 1 (23:15)	Unconscious, severe metabolic acidosis, transferred to ICU
D 2 (00:30)	Mechanical ventilation, CRRT (CVVHDF mode)
D 4 (09:30)	Basic stabilization of the internal environment, discontinued CRRT, blood culture with *A veronii*, switched to sensitive antibiotics
D 8	Infection indicators were controlled, body temperature normalized, blood culture negative again
D 11–14	CRRT again (CVVHDF mode), and transfusion of red blood cell suspension and fresh frozen plasma
D 18	The family gave up treatment and was automatically discharged

CRRT = continuous renal replacement therapy, CVVHDF = continuous veno-venous hemodiafiltration, ICU = intensive care unit.

## 3. Discussion

Patients with metformin-associated lactic acidosis (MALA)^[18,19]^ lack a specific clinical presentation; gastrointestinal symptoms may be the main symptoms, such as nausea, vomiting, bloating, and diarrhea.^[20]^ Most patients had disruption of lactate metabolism as a result of large oral doses of metformin over a short-period of time, but in this case, the patient did not. In conjunction with laboratory test results on admission, the patient was in a significant state of infection. When the patient presented with symptoms such as vomiting, the continued loss of fluids and lack of appropriate fluid replacement due to loss of appetite led to a lack of body volume, further aggravating the state of hypoxia and acidosis of the internal environment. Acidosis was considered to have been triggered by the infection, and subsequent blood cultures confirmed a specific *A veronii* infection. This patient had obvious nausea and vomiting symptoms before admission, consistent with gastroenteritis.^[5]^

Some studies have shown that although *A veronii* responds well to antibiotics,^[6,7,9]^ as the least common group of *Aeromonas* spp. in clinical practice, it has been considered in recent years as one of the causative agents of medically relevant bacteremia, especially in immunocompromised patients.^[1,10]^ Additionally, it may be closely associated with high mortality rates, cancer, and shock.^[5]^ In this case, the sensitive antibiotics were changed as soon as *A veronii* was detected. After the administration of these antibiotics, infection indicators such as white blood cell count, procalcitonin, and body temperature decreased. The internal environment has been relatively stable since dialysis, and it is considered that the main reason for the poor control of the infection is still related to the lack of clinical experience with *A veronii*, and that relying solely on drug sensitivity tests to guide clinical use is inadequate. The patient also showed signs of renal insufficiency and heart failure, and there were multiple uncertainties that interfered with the effectiveness of antibiotic therapy.

The current clinical work-up is increasing in the case of elderly patients who, because of a combined long history of chronic disease, require timely correction when the internal environment is suddenly disturbed. However, clinical work often does not achieve the expected speed, and with some causes due to severe or specific pathogenic infections, blood cultures take 3 to 5 days with a degree of lag and clinical judgment cannot be made in time, resulting in accelerated disease progression and reduced long-term outcomes for the patient. This phenomenon is becoming increasingly common in developing countries. Diabetes is second only to hypertension as a chronic disease in China, with a large number of patients requiring long-term oral hypoglycemic and antihypertensive medication. As the body’s resistance declines with age, these patients may suffer from serious internal environmental disturbances when the seasons change and other factors that increase the risk of infection. In our case, it was caused by a rare pathogenic bacterium. There is insufficient clinical knowledge and experience in the treatment of this type of *A veronii*, and when we look back at the literature on this type of bacterium,^[10,13,21]^ most reports are on infections in animals. In this case, some initial efficacy was observed, and the internal environment was corrected after dialysis treatment. However, the etiology was not clear, and the patient was in poor physical condition, with subsequent recurrent fever and inability to get off the machine successfully until the family finally gave up treatment.

Patients with lactic acidosis have low morbidity, poor prognosis, and high morbidity and mortality rates.^[19,22]^ Case reports that have been published describing MALA are mostly retrospective. There is no consensus regarding the diagnostic criteria for MALA in clinical practice.^[18,22]^ Therefore, it is extremely important for clinicians, especially emergency physicians to improve the diagnosis of acidosis. Early intervention is particularly important in cases of MALA, which may be caused by an underlying infection. As the clinical search for the causative organism takes several days, the early stages of the disease are critical and rapidly changing, requiring early intervention to avoid critical illness. This reminds clinicians that once lactic acidosis has been judged, the possibility of infection should be fully considered and sensitive antibiotics should be administered as early as possible. The emergence of these uncommon cases of *A veronii* also reminds us of previously rare bacterial infections. There are even clinical cases of reported fungal infections that suggest the need for antifungal drugs in addition to antibiotics in some cases, but the progression of disease severity in the patient often limits the large envelope of drugs; therefore, the use of multiple antibiotics plus antifungal or antiviral drugs at the outset of clinical work remains to be considered.

## Acknowledgements

The authors are grateful to the patient for providing permission to share the medical information.

## Author contributions

**Conceptualization:** Changxue Wu.

**Data curation:** Yu Xia.

**Formal analysis:** Yu Xia.

**Visualization:** Xiaofeng Zhu.

**Writing – original draft:** Yu Xia, Xiaofeng Zhu.

**Writing – review & editing:** Changxue Wu.
